# Temporal trends in the prescription of traditional Japanese herbal (Kampo) medicines to pregnant women: analysis of an administrative hospital database in Japan

**DOI:** 10.1186/s40780-025-00515-5

**Published:** 2025-12-24

**Authors:** Daisuke Kikuchi, Taku Obara, Shiro Hatakeyama, Risa Josaka, Misaki Tokunaga, Kouji Okada, Yuriko Murai

**Affiliations:** 1 Division of Community Medicine and Pharmacy, Faculty of Pharmaceutical Sciences, Tohoku Medical and Pharmaceutical University, 4-4-1 Komatsushima, Aoba-ku, Sendai, Miyagi 981-8558 Japan; 2 Department of Pharmacy, Tohoku Medical and Pharmaceutical University, Hospital 1-12-1, Fukumuro, Miyagino-ku, Sendai, Japan; 3 Department of Molecular Epidemiology, Tohoku University Graduate School of Medicine, 2-1, Seiryo-machi, Aoba-ku, Sendai, Japan; 4 Division of Preventive Medicine and Epidemiology, Tohoku University Tohoku Medical Megabank Organization, 2-1, Seiryo-machi, Aoba-ku, Sendai, Japan; 5 Department of Pharmaceutical Sciences, Tohoku University Hospital, 1-1, Seiryo-machi, Aoba-ku, Sendai, Japan; 6 Division of Clinical Pharmaceutics and Pharmacy Practice, Tohoku Medical and Pharmaceutical University, 1-12-1, Fukumuro, Miyagino-ku, Sendai, Japan; 7 Division of Clinical Pharmaceutics, Faculty of Pharmaceutical Sciences, Tohoku Medical and Pharmaceutical University, 4-4-1 Komatsushima, Aoba- ku, Sendai, Japan

**Keywords:** Administrative data, Herbal medicine, Kampo medicine, Pregnant women, Prescription

## Abstract

**Background:**

Japanese traditional (Kampo) medicines are commonly prescribed in clinical practice, with increasing evidence supporting their use during pregnancy. The efficacy and safety of Kampo medicines during pregnancy have increasingly been studied; however, evidence in support of these medicines is inadequate. Thus, we conducted a temporal trend analysis of Kampo medicine prescriptions to determine the Kampo medicines for which further safety evidence is required.

**Methods:**

Administrative data from pregnant Japanese women who visited acute-care diagnosis procedure combination hospitals between January 1, 2014, and December 31, 2023, were used in this study. Therapeutic categories related to the Kyoto Encyclopedia of Genes and Genomes D numbers 5100 and 5200 were defined as target Kampo medicines. Annual prescription trends were calculated as proportions. Temporal trends in the proportion of prescriptions for each Kampo medicine were assessed using the Cochran–Armitage trend test. Statistical significance was set at *p* < 0.05.

**Results:**

Between 2014 and 2023, the proportion of Kampo medicine prescriptions increased significantly from 12.0% to 13.6% (*p* < 0.001). As of 2023, *tokishakuyakusan* (2.9%) was the most prescribed medication, followed by *kakkonto* (2.4%) and *daikenchuto* (2.0%). From 2014 to 2023, the proportions of *tokishakuyakusan* (3.3% to 2.9%) and *kakkonto* (2.4% to 2.4%) prescriptions showed no significant temporal changes (*p* = 0.07 and 0.36, respectively). In contrast, the proportion of *daikenchuto* prescriptions increased significantly from 0.8% to 2.0% (*p* < 0.001).

**Conclusion:**

The primary prescribed Kampo medicines were those with established safety evidence for use in pregnant women. The proportion of Kampo medicine prescriptions for pregnant women in Japan has increased over time, with *tokishakuyakusan* being the most prescribed during the study period.

**Supplementary Information:**

The online version contains supplementary material available at 10.1186/s40780-025-00515-5.

## Background

Herbal medicines are used during pregnancy to manage various health conditions, with an estimated global prevalence of 28.9%, depending on regional and cultural differences [1]. Kampo, a traditional Japanese herbal medicine originating from traditional Chinese remedies, is occasionally used for pharmacotherapy in Japan [2–6]. Kampo medicines combine multiple herbs with complementary therapies and are commonly prescribed under the national health insurance system for pregnancy-related discomforts [7]. Medical education in Japan has increasingly incorporated Kampo medicine. The Model Core Curriculum for Medical Education (2010 and 2016 editions) requires students to understand the characteristics, indications, and pharmacological actions of Kampo medicines, including their relevance to specific populations such as pregnant women [8].

Kampo medicines such as *shoseiryuto*, *kakkonto*, and *tokishakuyakusan* have been used to address health concerns in pregnant women [4, 5] and are indicated for symptoms that tend to complicate pregnancy. *Shoseiryuto* is used as a cough suppressant and watery nasal discharge, *kakkonto* as a cold remedy, and *tokishakuyakusan* as a remedy for anemia and various illnesses during pregnancy. A survey of 19,220 pregnant women in the Tohoku Medical Megabank Project Birth and Three-Generation Cohort Study [4] showed that 4.5% of participants used Kampo medicines from diagnosis to 12 weeks of gestation, and 4.5% continued use afterward. Additionally, *shoseiryuto* was the most used Kampo medicine from diagnosis to 12 weeks of gestation and beyond. Similarly, a study using the JMDC claims database confirmed *shoseiryuto* as the most frequently prescribed Kampo medicine for pregnant women, followed by *tokishakuyakusan* and *kakkonto* [5]. Reports on the safety of Kampo medicines in the first pregnancy trimester [9, 10] did not find significant associations between the use of medicines containing ephedra or rhubarb rhizome and the risk of major congenital malformations [9, 10]. Thus, evidence regarding the safety of Kampo medicines in pregnant women is gradually accumulating.

Because the safety of Kampo medicines and the proportion of Kampo prescriptions among pregnant women have only been observed for specific periods, prescription trends over time need to be investigated to determine whether use of Kampo medicines in pregnant women is consistently safe. Moreover, evidence that supports the use of these medicines is inadequate. Therefore, we conducted a temporal trend analysis to determine Kampo medicines requiring further safety evidence.

## Materials and methods

### MDV analyzer and database

This retrospective survey utilized MDV analyzer (Medical Data Vision Co., Ltd., Tokyo, Japan) [11] and an anonymized administrative hospital database compiled by Medical Data Vision Co., Ltd., containing inpatient and outpatient records from Japanese acute-care hospitals. Data from 49.2 million patients, including deceased individuals, across 532 acute-care diagnosis procedure combination hospitals were included as of the maintenance endpoint in November 2024 [12].

### Study population

As MDV analyzer cannot directly identify pregnant women, patients with International Classification of Diseases, tenth revision (ICD-10), codes associated with pregnancy were classified as pregnant women [13]. These codes were derived from a previous study [14]. Women with pregnancy- and delivery-associated ICD-10 codes were included, while those with ICD-10 codes explicitly indicating the postpartum period within the same month were excluded (Table 1). The study population consisted of patients aged 20–49 years who visited an acute diagnosis procedure combination hospital providing administrative data to the MDV database. The study period was from January 1, 2014, to December 31, 2023.

**Table 1:** ICD-10 codes used for data extraction in pregnant women

ICD-10 code	Classification
Included ICD-10 codes:	
O00–O08	Pregnancy with abortive outcome
O10–O16	Edema, proteinuria, and hypertensive disorders in pregnancy, childbirth, and puerperium
O20–O29	Other maternal disorders predominantly related to pregnancy
O30–O48	Maternal care related to the fetus, amniotic sac, and potential delivery complications
O60	Preterm labor and delivery
O61	Failed induction of labor
O62	Labor abnormalities
O63	Prolonged labor
O64	Obstructed labor caused by fetal malposition or malpresentation
O65	Obstructed labor due to maternal pelvic abnormality
O66	Other obstructed labor
O67	Labor and delivery complicated by intrapartum hemorrhage, unspecified
O68	Labor and delivery complicated by fetal stress [distress]
O69	Labor and delivery complicated by umbilical cord issues
O70	Perineal laceration during delivery
O71	Other obstetric trauma
O74	Anesthesia-related complications during labor and delivery
O75	Other unspecified complications of labor and delivery
O80–O84	Delivery
O94–O99	Other unspecified obstetric conditions
Z33	Incidental pregnancy
Z34	Management of a normal pregnancy
Z35	Management of a high-risk pregnancy
Z36	Antenatal screening
Excluded ICD-10 codes:	
O72	Postpartum hemorrhage
O73	Retained placenta and membranes without hemorrhage

ICD-10: International Classification of Diseases, 10th revision

### Definition of target Kampo medicine prescriptions

Therapeutic categories related to the Kyoto Encyclopedia of Genes and Genomes [15] D numbers 5100 and 5200 were defined as target Kampo medicines. Annual counts of prescribed Kampo medicines from 2014 to 2023 were tabulated to mitigate intra-year prescription-variability effects and extracted from Japanese administrative data in MDV analyzer.

### Evaluation metrics

Evaluation metrics included the proportion of Kampo medicine prescriptions and distribution of each prescribed Kampo medicine. As raw patient data could not be accessed, the prescription proportion was calculated using available data with the following formula:

Proportion of Kampo medicine prescriptions = (number of pregnant women who were prescribed Kampo medicines/total number of pregnant women) ×100.

The formula considers only whether or not Kampo medicine was prescribed, but not the dosage or duration.

*Daikenchuto* is prescribed for constipation during pregnancy [16] and the prevention of ileus after cesarean Section [17]. Since MDV analyzer selects patients on a monthly basis using ICD-10 codes, *daikenchuto* prescriptions were compared between all pregnant women and those who underwent cesarean sections.

### Statistical analysis

The proportions of Kampo medicine prescriptions from 2014 to 2023 were calculated and compared using the Cochran–Armitage trend test. Statistical significance was set at *p* < 0.05. All statistical analyses were conducted using R version 4.3.3 (R Foundation for Statistical Computing, Vienna, Austria) [18].

## Results

Between 2014 and 2023, 21 Kampo medicines with various effects were prescribed (Supplementary Table 1). The proportion of Kampo medicine prescriptions for pregnant women increased from 12.0% to 13.6% (*p* < 0.001); yearly proportions are shown in Table 2.

**Table 2:** Yearly proportion of top ten Kampo medicine prescriptions for pregnant women

Year	2014	2015	2016	2017	2018	2019	2020	2021	2022	2023	*p*
Pregnant women, n	56,077	54,908	53,292	52,111	51,462	49,456	45,981	45,042	44,629	42,878	
Prescriptions for any Kampo medicine, n (%)	6,735 (12.0)	6,719 (12.2)	6,192 (11.6)	6,137 (11.8)	6,436 (12.5)	6,554 (13.3)	5,670 (12.3)	5,686 (12.6)	5,598 (12.5)	5,841 (13.6)	< 0.001
*Tokishakuyakusan*, n (%)	1,875 (3.3)	1,808 (3.3)	1,482 (2.8)	1,565 (3.0)	1,466 (2.8)	1,605 (3.2)	1,488 (3.2)	1,494 (3.3)	1,310 (2.9)	1,259 (2.9)	0.07
*Kakkonto*, n (%)	1,320 (2.4)	1,364 (2.5)	1,408 (2.6)	1,269 (2.4)	1,323 (2.6)	1,250 (2.5)	1,125 (2.4)	1,100 (2.4)	1,033 (2.3)	1,049 (2.4)	0.36
*Daikenchuto*, n (%)	449 (0.8)	423 (0.8)	403 (0.8)	536 (1.0)	444 (0.9)	556 (1.1)	602 (1.3)	604 (1.3)	677 (1.5)	862 (2.0)	< 0.001
*Saireito*, n (%)	1,132 (2.0)	1,161 (2.1)	972 (1.8)	891 (1.7)	925 (1.8)	868 (1.8)	854 (1.9)	848 (1.9)	841 (1.9)	781 (1.8)	0.007
*Goreisan*, n (%)	314 (0.6)	336 (0.6)	334 (0.6)	397 (0.8)	463 (0.9)	591 (1.2)	474 (1.0)	565 (1.3)	597 (1.3)	678 (1.6)	< 0.001
*Shoseiryuto*, n (%)	983 (1.8)	973 (1.8)	931 (1.7)	876 (1.7)	1,010 (2.0)	828 (1.7)	449 (1.0)	415 (0.9)	379 (0.8)	484 (1.1)	< 0.001
*Shakuyakukanzoto*, n (%)	146 (0.3)	120 (0.2)	119 (0.2)	137 (0.3)	166 (0.3)	268 (0.5)	291 (0.6)	324 (0.7)	309 (0.7)	329 (0.8)	< 0.001
*Bakumondoto*, n (%)	565 (1.0)	562 (1.0)	535 (1.0)	481 (0.9)	534 (1.0)	429 (0.9)	197 (0.4)	149 (0.3)	203 (0.5)	269 (0.6)	< 0.001
*Hangekobokuto*, n (%)	154 (0.3)	188 (0.3)	184 (0.3)	156 (0.3)	207 (0.4)	205 (0.4)	214 (0.5)	221 (0.5)	219 (0.5)	234 (0.5)	< 0.001
*Shohangekabukuryoto*, n (%)	183 (0.3)	155 (0.3)	158 (0.3)	150 (0.3)	184 (0.4)	182 (0.4)	155 (0.3)	151 (0.3)	159 (0.4)	157 (0.4)	0.009

Each Kampo medicine is listed according to its proportion of prescriptions in 2023

The Cochran–Armitage trend test was used for statistical analysis

A p-value < 0.05 was considered statistically significant

Additionally, as of 2023, the number of prescriptions for three medicines exceeded 2% of the total: *Tokishakuyakusan* (2.4%) was the most prescribed medication, followed by *kakkonto* (2.4%) and *daikenchuto* (2.0%). From 2014 to 2023, the proportions of *tokishakuyakusan* (3.3% to 2.9%) and *kakkonto* (2.4% to 2.4%) prescriptions showed no temporal changes (*p* = 0.07 and 0.36, respectively). Conversely, the proportion of *daikenchuto* prescriptions increased from 0.8% to 2.0% (*p* < 0.001). *Tokishakuyakusan* remained the most prescribed throughout the study period.

Table 3 shows the yearly proportions of *daikenchuto* prescriptions among pregnant women with cesarean section-associated ICD-10 codes. In this subgroup, the proportion of *daikenchuto* prescriptions increased from 19.8% to 42.3% (*p* < 0.001).

**Table 3:** *Daikenchuto* prescriptions for pregnant women and those with an ICD-10 code related to Cesarean section

Year	2014	2015	2016	2017	2018	2019	2020	2021	2022	2023	*p*
Number of pregnant women with an ICD-10 code related to cesarean section	4,544	4,569	5,022	5,008	5,115	5,112	4,858	4,621	4,787	4,610	-
Pregnant women with an ICD-10 code related to cesarean section who were prescribed *daikenchuto*, n (%)	89 (2.0)	87 (1.9)	106 (2.1)	125 (2.5)	130 (2.5)	160 (3.1)	191 (3.9)	199 (4.3)	217 (4.5)	365 (7.9)	< 0.001
(number of pregnant women with an ICD-10 code related to cesarean section who were prescribed *daikenchuto*, n)/[total number of pregnant women who were prescribed *daikenchuto*, n (as shown in Table 2)] × 100 (%)	19.8	20.6	26.3	23.3	29.3	28.8	31.7	32.9	32.1	42.3	< 0.001

ICD-10: International Classification of Diseases, tenth revision

The Cochran–Armitage trend test was used for statistical analysis

A p-value of < 0.05 was considered statistically significant

## Discussion

Our findings indicate that prescriptions of Kampo medicines for pregnant women have increased over time. Prescribing *tokishakuyakusan*, *kakkonto*, and *daikenchuto* to pregnant women is common in clinical practice [4, 5, 16]. One contributing factor to this upward trend may be increasing evidence supporting the use of Kampo medicines [4, 5, 9, 10, 19] and updated clinical guidelines [20, 21]. Additionally, the model core curriculum for medical education requests students to be able to explain the characteristics, current use (2010 edition), indications, and pharmacological actions of Kampo medicines (2016 edition) [8]. These education activities and updated guidelines may have contributed to the safe prescription of Kampo medicines to pregnant women.

*Tokishakuyakusan* and *kakkonto* were consistently and frequently prescribed as Kampo medicines for pregnant women between 2014 and 2023, without significant temporal changes in prescription frequencies during this period. *Tokishakuyakusan*, a versatile Kampo medicine, is recognized for effectively treating pregnancy-related symptoms, including anemia, edema, and cold hands or feet, which, according to Kampo theory, are attributed to blood stasis and fluid imbalance [22]. Similarly, *kakkonto* is widely prescribed for its efficacy in treating early-stage cold symptoms and musculoskeletal discomfort, which are common during pregnancy [4, 5]. The development of new Kampo formulations specifically targeting these pregnancy-related conditions remains limited. Therefore, *tokishakuyakusan* and *kakkonto* face little competition in the prescription drug market. Although the integration of Kampo medicine into modern healthcare is expanding, these formulations remain specialized and are likely to be consistently prescribed in the long term.

The proportion of *daikenchuto* prescriptions for pregnant women increased over time. Pregnant women experience constipation more frequently, likely due to hormonal imbalances during pregnancy [23]. In a single-center prospective study, 7.5 g of *daikenchuto* was administered daily for 28 days to 20 pregnant women with constipation [16]. Significant decreases in constipation assessment scale scores were observed on day 28 after *daikenchuto* administration (*p* = 0.019). The treatment was most effective during the second trimester. We additionally observed an increased proportion of *daikenchuto* prescriptions in pregnant women with cesarean section-associated ICD-10 codes. Because *daikenchuto* is commonly prescribed for constipation during pregnancy [16] and to prevent ileus after cesarean Sect [17], the increase in *daikenchuto* consumption over time warrants careful attention.

This study has some limitations. First, we focused on the prescription of Kampo medicines to pregnant women, without using other information provided by MDV analyzer such as prescription dosage or duration. Second, the safety profiles of prescribed Kampo medicines with ingredients for which caution is advised during pregnancy (such as moutan bark, peach kernel) have not been assessed. Clarification of the safety profiles for these ingredients is particularly desired in the future. Third, the timing of pregnancy during which Kampo medicines were prescribed could not be determined using MDV analyzer. However, this is the first report of the temporal trends in the prescription status of individual Kampo medicines for pregnant women in Japan using administrative data.

## Conclusion

Kampo medicines with established safety evidence for use in pregnant women were primarily prescribed, with an increase in prescriptions over time. *Tokishakuyakusan* remained the most prescribed drug throughout the study period. Moreover, *daikenchuto* prescriptions significantly increased during the study period. While *daikenchuto* is effective in treating constipation during pregnancy and preventing ileus after cesarean section, it warrants closer scrutiny due to potential gastrointestinal side effects. Although prescription patterns often reflect medicines with established safety profiles, clinical practice may not always align with evolving evidence. Continuous monitoring of prescribing trends is thus essential to ensure safe use of Kampo medicines in pregnant women.

## Supplementary Information

Below is the link to the electronic supplementary material.

Supplementary Material 1

## Data Availability

No datasets were generated or analysed during the current study.

## Abbreviations

ICD-10: International Classification of Diseases, tenth revision
