# Supplementary material for: Temporal trends in the prescription of traditional Japanese herbal (Kampo) medicines to pregnant women: analysis of an administrative hospital database in Japan
Source: J Pharm Health Care Sci. 2025 Dec 24;11:112. doi: 10.1186/s40780-025-00515-5 (PMC12729618; doi:10.1186/s40780-025-00515-5)
Supplement: Supplementary file 1 — Supplementary Material 1 [file 40780_2025_515_MOESM1_ESM.docx]

**Supplementary Tables**

**Supplementary Table 1.** Effects listed in the package insert for Kampo medicines provided in Table 2 and Supplementary Table 2

| Kampo medicines | Effects listed in the package insert |
| --- | --- |
| *Bakumondoto* | Obstructive phlegm, bronchitis, and bronchial asthma. |
| *Boiogito* | Declined renal function, obesity, arthritis, dermatosis, edema, hyperhidrosis, and menstrual irregularity. |
| *Daikenchuto* | Abdominal cold feeling and pain accompanied by abdominal flatulence. |
| *Goreisan* | Edema, alcoholic hangover, diarrhea, nausea, vomiting, dizziness, and headache. |
| *Goshakusan* | Gastroenteritis, low back pain, nerve pain, joint pain, menstrual pain, headache, oversensitivity to cold, menopausal disorder, and common cold. |
| *Goshuyuto* | Headache and vomiting. |
| *Hainosankyuto* | Symptoms of purulence with a reddened swollen and painful lesion. |
| *Hangekobokuto* | Neurotic gastritis, feeling that a thing is clogged up in the throat, morning sickness, coughing, hoarseness, and insomnia. |
| *Hochuekkito* | Summer emaciation, reinforcement of physical strength after illness, loss of appetite, gastroptosis, cold, hemorrhoid, anal prolapse, uterine prolapse, impotence, and hyperhidrosis. |
| *Kakkonto* | Common cold, coryza, inflammatory diseases, shoulder stiffness, nerve pain in the upper body, and hives. |
| *Kamishoyosan* | Oversensitivity to cold, delicate constitution, menstrual irregularity, menstrual pain/dysmenorrhea, and menopausal disorder. |
| *Keishibukuryogan* | Inflammatory diseases such as endometritis, menstrual irregularity, menstrual pain/dysmenorrhea, menopausal disorder, contusion, and oversensitivity to cold. |
| *Kososan* | Symptoms in the early stage of common cold in nervous people with gastrointestinal weakness. |
| *Ninjin'yoeito* | Declined constitution after recovery from disease, fatigue and malaise, loss of appetite, perspiration during sleep, cold limbs, and anemia. |
| *Rikkunshito* | Functional decline of the stomach and intestines, loss of appetite, gastric pain, vomiting. |
| *Saireito* | Watery diarrhea, acute gastroenteritis, heat exhaustion, and edema. |
| *Shakuyakukanzoto* | Myalgia or arthralgia, gastric pain and abdominal pain accompanied by sudden muscle spasms. |
| *shohangekabukuryoto* | Morning sickness and vomiting associated with other diseases. |
| *Shoseiryuto* | Watery phlegm, watery nasal discharge, nasal congestion, sneezing, wheezing, coughing, lacrimation in the above diseases. |
| *Tokishakuyakusan* | Anemia, malaise, menopausal disorder, menstrual irregularity, menstrual pain/dysmenorrhea, palpitation, and diseases during pregnancy. |
| *Yokukansan* | Neurosis, insomnia, child night cry, and peevishness in children. |
| Kampo medicines are listed alphabetically. | |

**Supplementary Table 2.** Yearly proportion of non-top ten Kampo medicine prescriptions for pregnant women

| Year | 2014 | 2015 | 2016 | 2017 | 2018 | 2019 | 2020 | 2021 | 2022 | 2023 | p |
| --- | --- | --- | --- | --- | --- | --- | --- | --- | --- | --- | --- |
| Pregnant women, n | 56,077 | 54,908 | 53,292 | 52,111 | 51,462 | 49,456 | 45,981 | 45,042 | 44,629 | 42,878 |  |
| Prescriptions for any Kampo medicine, n (%) | 6,735 (12.0) | 6,719 (12.2) | 6,192 (11.6) | 6,137 (11.8) | 6,436 (12.5) | 6,554 (13.3) | 5,670 (12.3) | 5,686 (12.6) | 5,598 (12.5) | 5,841 (13.6) | < 0.001 |
| *Keishibukuryogan*, n (%) | 63 (0.1) | 63 (0.1) | 48 (0.1) | 59 (0.1) | 67 (0.1) | 81 (0.2) | 64 (0.1) | 110 (0.2) | 128 (0.3) | 146 (0.3) | < 0.001 |
| *Goshakusan*, n (%) | 1 (0.0) | 2 (0.0) | 0 (0.0) | 17 (0.0) | 97 (0.2) | 123 (0.2) | 84 (0.2) | 67 (0.1) | 88 (0.2) | 111 (0.3) | < 0.001 |
| *Yokukansan*, n (%) | 28 (0.1) | 37 (0.1) | 39 (0.1) | 68 (0.1) | 61 (0.1) | 66 (0.1) | 85 (0.2) | 90 (0.2) | 79 (0.2) | 95 (0.2) | < 0.001 |
| *Rikkunshito*, n (%) | 54 (0.1) | 46 (0.1) | 59 (0.1) | 85 (0.2) | 77 (0.1) | 82 (0.2) | 79 (0.2) | 112 (0.2) | 72 (0.2) | 71 (0.2) | < 0.001 |
| *Boiogito*, n (%) | 3 (0.0) | 2 (0.0) | 2 (0.0) | 3 (0.0) | 5 (0.0) | 11 (0.0) | 7 (0.0) | 25 (0.1) | 55 (0.1) | 55 (0.1) | < 0.001 |
| *Kamishoyosan*, n (%) | 18 (0.0) | 26 (0.0) | 25 (0.0) | 37 (0.1) | 48 (0.1) | 40 (0.1) | 41 (0.1) | 48 (0.1) | 51 (0.1) | 53 (0.1) | < 0.001 |
| *Hochuekkito*, n (%) | 35 (0.1) | 44 (0.1) | 39 (0.1) | 32 (0.1) | 33 (0.1) | 29 (0.1) | 47 (0.1) | 41 (0.1) | 36 (0.1) | 42 (0.1) | 0.02 |
| *Goshuyuto*, n (%) | 55 (0.1) | 34 (0.1) | 18 (0.0) | 16 (0.0) | 18 (0.0) | 42 (0.1) | 39 (0.1) | 24 (0.1) | 43 (0.1) | 37 (0.1) | 0.07 |
| *Ninjin'yoeito*, n (%) | 16 (0.0) | 6 (0.0) | 13 (0.0) | 11 (0.0) | 52 (0.1) | 106 (0.2) | 116 (0.3) | 72 (0.2) | 31 (0.1) | 37 (0.1) | < 0.001 |
| *Hainosankyuto*, n (%) | 3 (0.0) | 9 (0.0) | 7 (0.0) | 2 (0.0) | 0 (0.0) | 3 (0.0) | 9 (0.0) | 28 (0.1) | 23 (0.1) | 32 (0.1) | < 0.001 |
| *Kososan*, n (%) | 9 (0.0) | 13 (0.0) | 11 (0.0) | 8 (0.0) | 9 (0.0) | 13 (0.0) | 6 (0.0) | 27 (0.1) | 35 (0.1) | 24 (0.1) | < 0.001 |
| Each Kampo medicine is listed according to its proportion of prescriptions in 2023. | | | | | | | | | | | |
| Kampo medicines with a prescription proportion ≥ 0.1% in 2023 are included in the list for pregnant women. | | | | | | | | | | | |
| The Cochran–Armitage trend test was used for statistical analysis. | | | | | | | | | | | |
| A p-value <0.05 was considered statistically significant. | | | | | | | | | | | |
